# A Comparative Cross-Sectional Study of Stressors and Experiences Among Pediatric and General Hospital Nurses in the Post-COVID-19 Era

**DOI:** 10.7759/cureus.80379

**Published:** 2025-03-10

**Authors:** Vasiliki Georgousopoulou, Georgios Manomenidis, Aspasia Serdari, Pantelis Perdikaris, Maria Amanatidou, Maria Lavdaniti, Sotiria Loukidou, Vaios Kalatzis, Vasiliki Matziou

**Affiliations:** 1 Department of Nursing, Democritus University of Thrace, Alexandroupolis, GRC; 2 Department of Child and Adolescent Psychiatry, Democritus University of Thrace, Alexandroupolis, GRC; 3 Department of Nursing, School of Health Sciences, University of Peloponnese, Tripolis, GRC; 4 Department of Nursing, University General Hospital of Alexandroupolis, Alexandroupolis, GRC; 5 Department of Nursing, International Hellenic University, Thessaloniki, GRC; 6 Department of Nursing, General Hospital of Ptolemaida, Ptolemaida, GRC; 7 Department of Surgical Anatomy, General Hospital of Ptolemaida, Ptolemaida, GRC; 8 Department of Nursing, National and Kapodistrian University of Athens, Athens, GRC

**Keywords:** anxiety, burnout, covid-19, mental health, pediatric nurses

## Abstract

The COVID-19 pandemic profoundly impacted the mental and physical well-being of nurses, with distinct challenges faced by pediatric and general hospital nurses. This study aimed to compare the stressors and experiences of these two groups in Greece during and after the pandemic. A cross-sectional survey of 249 nurses (108 pediatric and 141 general) (response rate: 71.1%) was conducted using a 34-item questionnaire adapted from the Coronavirus Health and Impact Survey (CRISIS) tool. While the questionnaire did not include a single item labeled "stressors," it contained sections measuring workload, burnout, perceived emotional burden, exposure risks, and mental health impacts. These variables serve as indicators of stressors within the healthcare context. The findings revealed significant differences between the two groups. Previous research suggests that pediatric nurses experience unique stressors, including the dual burden of addressing the psychological needs of children while providing emotional support to anxious families. The need for child-centered communication and high-quality care further amplifies these demands. However, pediatric nurses demonstrated greater resilience, likely due to their emphasis on maintaining physical activity as a coping mechanism. On the other hand, general hospital nurses exhibited reduced social interactions and a higher degree of social isolation compared to pediatric nurses. Despite facing common challenges, the distinct stressors highlight the necessity for targeted interventions. Tailored support systems should address the specific psychological and physical demands of pediatric nurses while also mitigating burnout and enhancing resilience among general hospital nurses.

These findings underscore the importance of implementing specialized mental health programs and workplace policies designed to address the unique challenges faced by pediatric and general hospital nurses. By addressing the distinct stressors experienced by each group, such initiatives can enhance resilience, reduce burnout, and promote overall well-being.

## Introduction

The COVID-19 pandemic was declared a public health emergency in 2019 and represents one of the most significant international public health crises in modern history [1]. Studies have shown that epidemics and disease outbreaks, including but not limited to, the COVID-19 pandemic, can lead to drastic individual and psychosocial changes, often more significant than the immediate medical impact. Apart from the immediate biological impacts, the COVID-19 pandemic caused broad and long-lasting disruptions in daily life, posing significant challenges to populations’ psychological well-being [2]. High levels of anxiety, stress, and depression were also observed in nurses during the COVID-19 pandemic, leading to feelings of isolation, powerlessness, and exhaustion [3].

Nurses with higher exposure to COVID-19 patients’ care experienced more severe symptoms of post-traumatic stress disorder, generalized anxiety disorder, and major depressive disorder [4]. In a study conducted by Hofmeyer and Taylor, general care nurses experienced increased levels of stress, burnout, and mental exhaustion due to extended working hours, staffing shortages, and constant changes in safety protocols [5]. Evidence suggests that inadequate support and working conditions, exacerbated by the pandemic, contributed to a decrease in nurses' mental resilience and an increase in burnout rates [6]. Furthermore, the pandemic significantly disrupted nurses' health behaviors. A study by Joy et al. found that nurses reported reduced physical activity, poorer dietary choices, and irregular sleep patterns, which negatively impacted their overall mental health and resilience [7]. Tremayne and de Bourg noted that disrupted sleep and decreased exercise were closely linked to increased anxiety and lower psychological well-being in nurses during the pandemic [8]. These changes in daily routines further compounded the mental and emotional strain experienced by healthcare workers, underscoring the need for holistic support strategies that address both mental health and health behaviors.

Even though most nursing specialties faced the detrimental impact of the pandemic in their everyday practice, pediatric nurses, in particular, encountered unique stressors. These included addressing the mental health needs of children, who often exhibit psychological distress differently from adults, requiring tailored communication and emotional support strategies. Pediatric nurses had to manage not only the physical care of children but also the emotional strain of supporting anxious families and maintaining a child-friendly environment amid strict infection control protocols [9]. The need to provide developmentally appropriate care while coping with visitation restrictions, disrupted routines, and the emotional toll of isolating young patients added another layer of complexity and pressure [10]. Additionally, pediatric nurses had to manage the significant emotional distress of the parents of hospitalized children, whose anxiety and stress added to the nurses' workload and emotional burden. The study of Páll et al. noted that pediatric nurses’ psychological burden increased due to the dual support provided to both children and their parents [11]. Pediatric nurses faced the need to not only manage the mental health of children who experienced increased levels of anxiety and emotional distress but also had to provide empathy and support to parents who experienced uncertainty and fear. These additional responsibilities created a more complex care environment compared to that of adult patients. This complexity distinguished their experience from that of adult patient nurses, who managed similar mental health issues but without the added challenge of simultaneously supporting worried and anxious parents while navigating child-specific developmental and emotional needs. Pediatric nurses had to balance effective communication with both children and parents while addressing the children’s psychological distress, which often manifests differently than in adults [9,12]. The need for tailored emotional care, combined with visitation restrictions, increased their psychological burden and required specialized skills [10].

To the best of our knowledge, this is the first comparative study in Greece examining the psychological well-being, emotional responses, and lifestyle changes of general and pediatric nurses before and after the COVID-19 pandemic. While international studies have explored nurses' stressors during the pandemic, few have specifically compared how pediatric and general hospital nurses experienced changes in mental health, work-related challenges, and coping mechanisms [6,12]. The physical and mental health, work habits, and emotional distress, which serve as indicators of occupational stressors, were examined. By examining how pediatric and general hospital nurses were affected differently and identifying their specific stressors and needs, this research provides critical insights into how healthcare systems can tailor interventions to better support these groups. Understanding these differences is key to developing targeted mental health support and resilience-building strategies, ensuring that healthcare workers are better equipped in future crises. This study also offers guidance for improving workplace support and training programs to reinforce the well-being of nurses in similar situations.

## Materials and methods

A cross-sectional study was conducted in one pediatric and three general hospitals in Greece using a self-administered questionnaire between February 2022 and September 2022. A random sampling method was employed to ensure unbiased participant selection. The hospitals were chosen to provide a representative sample across different regions of Greece.

Participants were required to be 18 years or older, of any gender, racial or ethnic background, and health status. To be included, individuals had to self-identify as healthcare professionals and specifically as medical or nursing staff employed in the National Health System (NHS). This self-identification was further verified through targeted screening questions within the study to ensure the accuracy of the sample and prevent selection bias.

The selection of hospitals was designed to capture a geographically representative sample of healthcare workers across Greece, ensuring a more comprehensive understanding of the impact of the COVID-19 pandemic on both pediatric and general hospital nurses. The research team distributed information leaflets to nursing personnel across various departments. A total of 249 nurses voluntarily and anonymously completed the questionnaire, yielding a response rate of 71.1% (n = 249).

For this study, a 34-item scale was developed using questions from the Coronavirus Health and Impact Survey (CRISIS) questionnaire [13]. The CRISIS tool was designed to assess the impact of the COVID-19 pandemic on mental health and behavior in various international settings. It aims to understand pandemic-induced changes and identify factors affecting mental well-being.

The CRISIS tool incorporates standardized measurement instruments such as the Generalized Anxiety Disorder Scale (GAD-7), the Patient Health Questionnaire (PHQ-9) for depression, and the University of California, Los Angeles (UCLA) Loneliness Scale. It examines life changes related to social contact, living situations, food security, financial stress, COVID-19-related worries, and mood states based on emotional experiences in the past two weeks. The questionnaire covers several domains, including demographics, health status, marital status, exposure to the virus, and changes in life due to the pandemic. It also explores daily habits such as sleep patterns, physical activity, emotions, concerns, media consumption, and substance use.

Both the Baseline and Follow-Up versions of the CRISIS questionnaire were used, as the study was conducted toward the end of the pandemic. The Baseline Form includes information about life/habits before the pandemic and the last two weeks, while the Follow-Up Form assesses the same areas at set intervals (e.g., weekly or monthly). Permission to use the CRISIS questionnaire was obtained from its creators, and the tool was adapted for the Greek population.

The CRISIS questionnaire was adapted for the Greek population using the forward-backward translation method, ensuring linguistic and conceptual equivalence. Two independent bilingual experts translated the questionnaire from English to Greek, and a third reconciled discrepancies. A separate bilingual expert conducted a back-translation to English to verify accuracy. A panel of three healthcare professionals and two researchers reviewed the translation for content validity and cultural relevance.

Additionally, a pilot study with 15 nurses was conducted to assess the comprehensibility of the translated questionnaire. Minor linguistic refinements were incorporated based on their feedback. Since the questionnaire was well understood by the participants, it was used in the main study without further modifications.

The CRISIS questionnaire has demonstrated previous psychometric properties, including high internal consistency (Omega > 0.8) for domains like mood states and COVID-related worries, and good test-retest reliability (Intraclass Correlation Coefficient, or ICC = 0.79-0.87). The nurses were assured that their participation was both voluntary and anonymous. A detailed description of how confidentiality and anonymity would be protected was provided in a cover letter that accompanied the questionnaire.

The study received ethical approval from the Scientific Council - Ethics and Deontology Committee (Approval No. 507/19-11-2020), and all participants were informed about the study's objectives, as well as their right to withdraw at any time.

Statistical analysis

Data were analyzed using IBM SPSS Statistics for Windows, Version 22 (Released 2013; IBM Corp., Armonk, NY, USA). Descriptive statistics were used to summarize demographic data, presenting categorical variables as absolute (N) and relative frequencies (%), and continuous variables as means with standard deviations (SDs). The normality of continuous variables was assessed using the Kolmogorov-Smirnov and Shapiro-Wilk tests. The McNemar test was applied for paired categorical data to evaluate changes in health behaviors, mental well-being, and work-related stressors before and after the pandemic. Chi-square (χ²) tests were used to compare categorical variables between pediatric and general hospital nurses. Independent t-tests and Mann-Whitney U tests were conducted to compare means between the two groups, depending on the normality of distribution. Repeated measures analysis of variance (ANOVA) or Wilcoxon signed-rank tests (for non-normally distributed data) were used to analyze changes in nurses’ physical and mental health across different time points.

## Results

A total of 249 nurses participated in the study (108 worked in pediatric and 141 worked in general hospitals). The sample consisted predominantly of females (80%, or n = 200). The mean age of the participants was 42 (±9.45) years for males and 42 (±9.77) years for females. The majority had tertiary education (60.6%, or n = 151), were married (52%, or n = 129), and worked in the Intensive Care Unit (ICU) (34%, or n = 85) (Table 1).

**Table 1:** Demographic characteristics The table shows the demographic characteristics of the nurses participating in the study. Specifically, it shows the distribution of participants, mean age, educational level, marital status, and work department.

Variables	Ν	%
Nursing personnel		
Pediatric nurses	108	43
Nurses from general hospital	141	57
Age (years)	42 (±9.45)	
Level of education		
University education	28	11.6
Tertiary education	151	60.6
Postgraduate degree (PhD)/Masters degree	70	28.8
Family status		
Divorced	20	8
Married	129	52
Single	100	40
Working department		
ICU	85	34
COVID-19 Department	42	17
Internal Medicine Department	68	27
Cardiology Department	38	6
Surgical Department	11	5
Other departments (Special Infection Unit, Day Care Unit, Emergency Care Department)	5	2

Overall, the physical health of nurses significantly changed during the pandemic, with general hospital nurses experiencing a greater decline (p < 0.001). Before the pandemic, 31.2% (n = 44) of pediatric nurses rated their physical health as "very poor," compared to 8.3% (n = 9) of general hospital nurses. In the post-COVID era, this percentage decreased to 24.8% (n = 35) for pediatric nurses but increased to 24% (n = 26) for general hospital nurses. This suggests a greater deterioration in perceived physical health among general hospital nurses, while pediatric nurses experienced a shift across different health categories rather than a worsening in the "very poor" category specifically. 

Regarding mental health, both pediatric and general hospital nurses showed statistically significant differences in those who reported poor or moderate mental health before and after the pandemic (pediatric nurses: p = 0.021, general hospital nurses: p < 0.001, overall: p < 0.001). These results indicate that the pandemic had a significant negative impact on the mental health of nurses, regardless of hospital type, with a more pronounced statistical significance in general hospital nurses. Before the pandemic, both groups rated their mental health as "excellent" (63.8% (n = 90) for pediatric nurses and 65.7% (n = 71) for general hospital nurses). However, in the post-COVID era, there was a notable increase in nurses rating their mental health as "very poor" in both groups, with a greater rise among general hospital nurses (from 6.5% (n = 7) to 27% (n = 39)). In terms of sleep habits before and after the pandemic, there was a significant difference in bedtime routines, with general hospital nurses going to bed later than pediatric nurses (p = 0.005). Regarding weekend sleep duration, before and after the pandemic, the majority of nurses, regardless of hospital type, reported sleeping an average of six to eight hours on weekends (58.6%, or n = 61). However, among general hospital nurses, 20% (n = 34) reported sleeping only four to six hours, a statistically significant difference (p = 0.005). Pediatric nurses also reported significant changes in their perceived physical health before and after the pandemic (p = 0.002), with a notable decline and an associated shift in exercise routines and activity levels. In contrast, general hospital nurses did not show a statistically significant change in their perception of physical health. Regarding weekend sleeping habits, before and after the pandemic, all nurses, regardless of hospital, slept an average of six to eight hours on weekends (58.6%, or n = 61). However, compared to the two nursing categories, 20% (n = 34) of general hospital nurses slept four to six hours. The differences in sleep time on weekends were statistically significant (p = 0.005).

Both groups had altered sleep schedules, with pediatric nurses showing statistically significant changes in sleep duration before and after the pandemic (p = 0.039). Pediatric nurses reported significant changes in their physical health assessments before and after the pandemic (p = 0.002), with a noticeable shift in exercise routines and habits. General hospital nurses did not show a statistically significant change in physical health perception. Pediatric nurses, who exercised one to two times per week, showed a reduction in exercise frequency post-pandemic, as well as those not exercising at all. Although there was no statistically significant difference, there was a shift in the behavior of nurses who did not exercise before the pandemic, indicating a potential change influenced by pandemic conditions. General hospital nurses exhibited more anxiety post-pandemic compared to pediatric nurses (p < 0.001). Both groups displayed increased levels of moderate to high anxiety, but pediatric nurses showed resilience, with lower reported increases. Before the pandemic, general hospital nurses tended to rate their physical health more favorably than pediatric nurses, with more than half of them rating it as "excellent." In contrast, a significantly higher percentage of pediatric nurses rated their health as "very poor." After the pandemic, however, the health ratings among pediatric nurses changed. The percentage of those who rated their health as "very poor" decreased to 24.8% (n = 35), while 34% (n = 48) rated their health as "moderate." The percentage of pediatric nurses who rated their health as "excellent" remained unchanged at 41.1% (n = 58) (Table 2).

**Table 2:** Differences between Pediatric Nurses and General Hospital Nurses before and after the pandemic in general emotions, psychological well-being, and lifestyle changes This table compares the habits, health, and psychosocial status of Pediatric and General Hospital Nurses before and after the COVID-19 pandemic. It assesses their physical and mental health, sleep hours, stress and well-being levels, sleep and exercise habits, alcohol and smoking consumption, and general sense of happiness and comfort. The results show differences between the two groups in several areas, with significant changes in their daily lives during the pandemic. The McNemar test was used to assess whether there were significant changes in the responses of pediatric nurses and general hospital nurses before and during the pandemic. A statistically significant McNemar p-value (<0.05) indicates that there was a meaningful shift in responses over time within a group. For comparisons between Pediatric and General Hospital Nurses, we used the McNemar test to evaluate whether differences in responses were statistically significant at each time point. Some p-values indicate no significant differences (>0.05), meaning that responses remained relatively stable over time.

Questions	Pediatric Nurses		General Hospital Nurses		p-value within Pediatric Nurses and General Hospital Nurses
How did you assess your physical health before the COVID-19 pandemic?	N	%	N	%	Pediatric Nurses, p = 0.002; General Hospital Nurses, p < 0.001
Very bad	44	31.2	9	8.3	
Average	39	27.6	41	38	
Extremely good	58	41.1	58	53.7	
Pediatric Nurses vs. General Hospital Nurses; McNemar = 1.0					
How do you assess your physical health in the last two weeks?	N	%	N	%	
Very bad	35	24,8	26	24	
Average	48	34	56	51.8	
Extremely good	58	41.1	26	24	
Pediatric Nurses vs. General Hospital Nurses; McNemar <0.001					
How did you assess your mental health before the COVID-19 pandemic?	N	%	N	%	Pediatric Nurses, p = 0.021; General Hospital Nurses, p < 0.001
Very bad	8	5.6	7	6.5	
Average	35	24.8	30	27.7	
Extremely good	90	63.8	71	65.7	
Pediatric Nurses vs. General Hospital Nurses; McNemar <0.001					
How do you assess your mental health in the last two weeks?	N	%	N	%	
Very bad	24	17	39	27	
Average	33	23.4	39	27	
Extremely good	84	59.5	67	46.2	
Pediatric Nurses vs. General Hospital Nurses; McNemar <0.001					
Approximately what time did you go to bed on weekdays before the start of the pandemic?	N	%	N	%	Pediatric Nurses, p = 0.07; General Hospital Nurses, p = 0.005
Before 9 pm	1	0.9	4	0.2	
9 pm to 11 pm	17	16.3	26	18	
11 pm to 1 am	68	76.9	63	42.3	
After 1 am	18	17.3	18	26	
Pediatric Nurses vs. General Hospital Nurses; McNemar <0.001					
Approximately what time did you go to bed on weekdays the last two weeks?	N	%	N	%	
Before 9 pm	2	0.3	1	0.6	
9 pm to 11 pm	26	25	26	18	
11 pm to 1 am	54	52	21	15	
After 1 am	21	20.2	97	66.4	
Pediatric Nurses vs. General Hospital Nurses; McNemar <0.001					
On average, how many hours did you sleep on weekday nights before the start of the pandemic?	N	%	N	%	Pediatric Nurses, p = 0.039; General Hospital Nurses, p < 0.001
4-6 hours	18	17.3	78	53.7	
6-8 hours	63	60.5	34	23.4	
8-10 hours	16	15.3	27	18.6	
Under 4 hours	3	0.2	2	0.1	
Up 10 hours	4	0.3	4	0.2	
Pediatric Nurses vs. General Hospital Nurses; McNemar <0.005					
On average, how many hours of sleep did you get on weekend nights in the last two weeks?	Ν	%	Ν	%	
4-6 hours	28	27	34	20	
6-8 hours	61	58.6	78	84	
8-10 hours	11	10.5	25	29	
Under 4 hours	2	0.1	4	1	
Up 10 hours	2	0.1	4	11	
Pediatric Nurses vs. General Hospital Nurses; McNemar <0.005					
How many days a week did you exercise (including vigorous walking) for at least 30 minutes to, for example, induce faster twitching or breathing before the pandemic started?	N	%	N	%	Pediatric Nurses, p = 0.182; General Hospital Nurses, p = 0.038 and Pediatric Nurses, p = 0.075; General Hospital Nurses, p = 0.001
1-2 days	42	43.2	70	48.2	
3-4 days	27	26	49	33.7	
5-6 days	5	4	2	0.1	
Every day	11	10.5	3	0.2	
No one	19	18.2	21	14.4	
Pediatric Nurses vs. General Hospital Nurses; McNemar = 0.075					
How many days a week did you exercise (including intense exercise)? walking) for at least 30 minutes to, for example, cause faster twitching or breathing in the last two weeks?	N	%	N	%	
1-2 days	42	40.3	72	49.6	
3-4 days	11	10.5	21	14.4	
5-6 days	7	0.6	3	0.2	
Every day	18	17.3	4	0.3	
No one	26	18	45	31	
Pediatric Nurses vs. General Hospital Nurses; McNemar = 0.650					
How many days a week did you go out of the house (parks, squares, outdoors, entertainment, etc.) before the start of the pandemic?	N	%	N	%	
1-2 days	42	40.3	47	32.4	
3-4 days	28	27	49	33.7	
5-6 days	13	12	9	6	
Every day	15	14.4	7	4	
No one	6	5	3	2	
Pediatric Nurses vs. General Hospital Nurses; McNemar <0.0001					
How many days a week did you go out of the house (parks, squares, outdoors, entertainment, etc.) in the last two weeks?	N	%	N	%	
1-2 days	32	30.7	59	40.6	
3-4 days	34	23.4	47	32.4	
5-6 days	17	11.7	23	15.8	
Every day	10	6	2	1	
No one	11	7	14	9	
Pediatric Nurses vs. General Hospital Nurses; McNemar <0.0001					
To what extent were you generally concerned before the pandemic started?	N	%	N	%	Pediatric Nurses, p = 0.911; General Hospital Nurses, p < 0.0001
Follow	20	19.2	7	4	
Μoderate	78	75	133	91.7	
Very	6	5	5	3	
Pediatric Nurses vs. General Hospital Nurses; McNemar = 0.911					
To what extent were you generally concerned in the last two weeks?	N	%	N	%	
Follow	20	19.2	4	2	
Μoderate	79	76	107	73.7	
Very	5	4	34	23.4	
Pediatric Nurses vs. General Hospital Nurses; McNemar <0.0001					
How sad or happy were you (for whatever reason) before the start of the pandemic?	N	%	N	%	Pediatric Nurses, p = 0.002; General Hospital Nurses, p < 0.0001
Μoderately sad	10	9	63	43.4	
Moderately happy	25	24	44	30.3	
Νeutral	41	39.4	42	28.9	
Very sad	4	3	2	1	
Very happy	24	23	24	16.5	
Pediatric Nurses vs. General Hospital Nurses; McNemar = 0.002					
How sad or happy have you been (for whatever reason) in the last two weeks?	N	%	N	%	
Μoderately sad	14	13.4	41	28.2	
Moderately happy	27	26	41	28.2	
Νeutral	48	46.1	47	32.4	
Very sad	6	5	5	3	
Very happy	9	8	11	7	
Pediatric Nurses vs. General Hospital Nurses; McNemar = 0.461					
How much were you able to enjoy your normal activities before the pandemic started?	N	%	N	%	Pediatric Nurses, p < 0.0001; General Hospital Nurses, p < 0.0001
Follow	4	3	2	1	
Μoderator	35	33.6	40	27.5	
Very much	65	62.5	103	71	
Pediatric Nurses vs. General Hospital Nurses; McNemar <0.0001					
How much have you been able to enjoy your usual activities in the last two weeks?	N	%	N	%	
Follow	9	8	4	2	
Μoderator	65	62.5	92	63.4	
Very much	30	28.8	49	33.7	
Pediatric Nurses vs. General Hospital Nurses; McNemar = 0.289					
How relaxed or stressed were you (for whatever reason) before the pandemic started?	N	%	N	%	Pediatric Nurses, p = 0.188; General Hospital Nurses, p = 0.323
Μoderately anxious	24	23	30	20,6	
Μoderately loose	25	24	25	17.2	
Νeutrality	38	36.5	56	38.6	
Very anxious	3	2	28	19.3	
Very loose	14	13.4	6	4	
Pediatric Nurses vs. General Hospital Nurses; McNemar = 0.188					
How relaxed or stressed have you been (for whatever reason) in the last two weeks?	N	%	N	%	
Μoderately anxious	31	29.8	30	20.6	
Μoderately loose	17	16.3	40	27.5	
Νeutrality	37	35.5	56	38.6	
Very anxious	7	6	13	8	
Very loose	12	11.5	6	4	
Pediatric Nurses vs. General Hospital Nurses; McNemar = 0.017					
How nervous (or anxious) were you (for whatever reason) before the pandemic started?	N	%	N	%	Pediatric nurses, p = 0.146; General Hospital Nurses, p = 0.080
Νot at all nervous or anxious	27	26	39	26.8	
Moderately nervous or anxious	67	64.4	100	69	
Very nervous or anxious	10	9	6	4	
Pediatric Nurses vs. General Hospital Nurses; McNemar = 0.146					
How nervous (or anxious) have you been (for whatever reason) in the last two weeks?	N	%	N	%	
Νot at all nervous or anxious	24	23	39	26.8	
Moderately nervous or anxious	68	65.3	88	60.6	
Very nervous or anxious	12	11.2	18	12.4	
Pediatric Nurses vs. General Hospital Nurses; McNemar = 0.542					
How tired (or exhausted) were you (for whatever reason) before the start of the pandemic?	N	%	N	%	Pediatric Nurses, p = 0.359; General Hospital Nurses, p = 0.404
Νot at all tired	6	5	10	6	
Moderately tired	81	77.8	106	73.1	
Very tired	17	16.3	29	20	
Pediatric Nurses vs. General Hospital Nurses; McNemar = 0.359					
How tired (or exhausted) have you been (for whatever reason) in the last two weeks?	N	%	N	%	
Νot at all tired	5	4	7	4	
Moderately tired	63	60.5	58	40	
Very tired	36	34.6	80	55	
Pediatric Nurses vs. General Hospital Nurses; McNemar = 0.264					
How lonely were you before the pandemic started?	N	%	N	%	Pediatric Nurses, p = 0.492; General Hospital Nurses, p = 0.553
No loneliness at all	57	54.8	76	52.4	
Moderate loneliness	43	41.3	64	44.1	
Excessive loneliness	4	3	5	3	
Pediatric Nurses vs. General Hospital Nurses; McNemar = 0.492					
How much loneliness have you felt in the last two weeks?	N	%	N	%	
No loneliness at all	57	54.8	66	41	
Moderate loneliness	40	38.4	66	41	
Excessive loneliness	7	6	13	9	
Pediatric Nurses vs. General Hospital Nurses; McNemar = 0.148					
Watched TV or digital media (e.g., Netflix, YouTube) or "surf" the internet before the start of the pandemic?	N	%	N	%	Pediatric Nurses, p = 0.176; General Hospital Nurses, p = 0.151
1-3 hours a day	50	48	74	51	
4-6 hours a day	5	4	20	13.7	
None	9	8	10	6	
Less than 1 hour per day	37	35.5	32	22	
More than 6 hours per day	3	2	9	6	
Pediatric Nurses vs. General Hospital Nurses; McNemar = 0.176					
Watched TV or digital media (e.g., Netflix, YouTube) or "surf" the internet in the last two weeks	N	%	N	%	
1-3 hours a day	51	49	69	47.5	
4-6 hours a day	8	7	30	20.6	
None	8	7	4	2	
Less than 1 hour per day	34	32.6	34	23.4	
More than 6 hours per day	4	3	3	2	
Pediatric Nurses vs. General Hospital Nurses; McNemar = 0.599					
Use of alcohol before the pandemic started?	N	%	N	%	Pediatric Nurses, p = 0.507; General Hospital Nurses, p = 0.535
Not at all	29	27.8	49	3.7	
Several times per week	10	9	12	8	
Once a week	27	35.5	42	28.9	
Once a day	8	7	4	2	
Infrequently (less than once a week)	30	28.8	38	26.2	
Pediatric Nurses vs. General Hospital Nurses; McNemar = 0.507					
Use of alcohol in the last two weeks	N	%	N	%	
Not at all	39	26.8	60	41.3	
Several times per week	12	8	20	13.7	
Once a week	52	35.8	24	16.5	
Once a day	4	2	6	4	
Infrequently (less than once a week)	38	26.2	35	24	
Pediatric Nurses vs. General Hospital Nurses; McNemar = 0.081					
Tobacco products or cigarettes before the pandemic started?	N	%	N	%	Pediatric Nurses, p = 0.372; General Hospital Nurses, p = 0.448
10-20 times per day	7	6	8	5	
2-5 times per day	8	7	20	13	
6-10 times per day	10	9	8	5	
Several times per week	2	1	6	4	
Not at all; I've stopped smoking	14	13.4	35	24.1	
Not at all; I've never smoked	46	44.2	53	36.5	
Once per week	6	5	4	2	
Once per day	1	0.9	3	2	
Over 20 times a day	2	1	3	2	
Infrequently (less than once a week)	8	7	5	36	
Pediatric Nurses vs. General Hospital Nurses; McNemar = 0.372					
Tobacco products or cigarettes in the last two weeks	N	%	N	%	
10-20 times per day	5	5	10	6	
2-5 times per day	7	6	16	11	
6-10 times per day	6	5.7	9	6	
Several times per week	3	2	8	6	
Not at all; I've stopped smoking	24	23	17	11	
Not at all; I've never smoked	48	46.1	73	50.3	
Once per week	1	0.9	2	1	
Once per day	2	1	1	1	
Over 20 times a day	2	1	3	2	
Infrequently (less than once a week)	6	5	6	4	
Pediatric Nurses vs. General Hospital Nurses; McNemar = 0.081					
How many days a week did you exercise (including vigorous walking) for at least 30 minutes to, for example, induce faster twitching or breathing before the pandemic started?	N	%	N	%	Pediatric Nurses, p = 0.856; General Hospital Nurses, p = 0.866
1-2 days	48	46.1	71	48.9	
3-4 days	27	18.6	48	33.1	
5-6 days	5	4	2	1	
Every day	5	4	3	2	
No one	19	13.1	21	14.4	
Pediatric Nurses vs. General Hospital Nurses; McNemar = 0.856					
How many days a week did you exercise (including vigorous walking) for at least 30 minutes to, for example, induce faster twitching or breathing in the last two weeks?	N	%	N	%	
1-2 days	42	40.3	42	28.9	
3-4 days	16	15.3	21	14.4	
5-6 days	7	6	3	2	
Every day	2	1	4	3	
No one	36	34.6	45	31	
Pediatric Nurses vs. General Hospital Nurses; McNemar = 0.870					

## Discussion

The purpose of this study was to compare the differences in psychological well-being, work-related experiences, and lifestyle changes among nurses in pediatric and general hospitals during the COVID-19 pandemic. Regarding physical health, the study found that general hospital nurses experienced a significant deterioration, whereas pediatric nurses showed some fluctuations in self-rated physical health. While the percentage of pediatric nurses rating their health as "very poor" decreased post-pandemic, the overall shift in physical health perceptions within this group suggests a complex pattern of change. In contrast, general hospital nurses reported a notable decline in physical health (p < 0.001) [8,14]. Nurses reported issues such as chronic fatigue, musculoskeletal pain, and decreased overall well-being, largely attributed to the intense demands of healthcare settings and insufficient recovery time [14,15]. In the past two weeks, only 24% (n = 26) of general hospital nurses and 24.8% (n = 35) of pediatric nurses rated their physical condition as "excellent." Additionally, the majority of nurses from both groups reported their physical condition as "moderate" or "poor," with a statistically significant decline (p < 0.001). These findings highlight how the stress and physical demands of the pandemic negatively affected the physical fitness of nurses across all specialties. While both groups experienced physical deterioration, pediatric nurses faced additional challenges as they adapted to new and more demanding environments. During the COVID-19 pandemic in Greece, pediatric nurses were often transferred to general hospitals, exposing them to more physically demanding settings and increasing their workload. These adjustments frequently required them to manage unfamiliar protocols, address children's psychological needs, and support anxious families, further exacerbating their physical strain and contributing to the overall decline in physical fitness.

Regarding sleep habits, our study found a significant difference in sleep patterns before and after the pandemic, with general hospital nurses going to bed earlier and sleeping more hours on weekends compared to pediatric nurses. A possible explanation for this difference could be the distinct nature of work environments and stressors faced by each group. General hospital nurses often engage in physically demanding tasks, such as lifting, frequent patient mobilization, and handling critical emergency situations, which may lead to greater physical exhaustion and an increased need for sleep recovery. In contrast, pediatric nurses experienced emotionally demanding stressors related to supporting children through illness and addressing the concerns of their families. This emotional burden may not result in the same level of physical exhaustion but could lead to increased mental and emotional stimulation, making it more difficult to relax and maintain stable sleep schedules. While the study by Kim-Godwin et al. [16] evaluated sleep patterns among Norwegian nurses and noted an increase in those sleeping less than six hours per night, there is still limited literature directly comparing the sleep habits of pediatric and general hospital nurses concerning poor sleep quality. Our findings suggest that the type of strain - whether physical or emotional - may have influenced sleep patterns differently between these groups. This finding aligns with a systematic review by Robba et al., which showed a widespread decline in nurses’ mental health during the COVID-19 pandemic [17]. The review found that nurses exhibited high rates of anxiety, depression, and psychological distress due to factors such as fear of infection, the risk of transmitting the virus to family members, and excessive workload. Specifically, pediatric nurses reported relatively lower levels of anxiety compared to their counterparts in general hospitals. This difference may be attributed to the fact that pediatric nurses were not typically involved in high-risk, life-threatening situations as commonly observed in general hospitals during the pandemic. However, pediatric nurses faced a distinct set of stressors. They were frequently reassigned to unfamiliar, understaffed departments, which not only posed challenges to their clinical practice but also had a significant impact on their mental and physical well-being. Increased workload, lack of preparation for new roles, and the emotional strain of caring for children during such a stressful period contributed to the unique stress experienced by pediatric nurses. Regarding higher anxiety levels among general hospital nurses, the meta-analysis by Al Maqbali et al. indicates that general hospital nurses had higher anxiety levels post-pandemic due to long working hours, fear of infection, and continuous exposure to critically ill patients [18]. Although this analysis does not directly compare pediatric nurses, it highlights unique stressors that were more intense in general hospitals. Conversely, pediatric nurses also experienced anxiety during the pandemic, but their anxiety levels were generally lower. This is consistent with the findings of De Kock et al., who confirmed that healthcare workers, in general, exhibited increased mental health issues during the pandemic [19].

In our study, all nurses, regardless of hospital type, showed reduced physical activity levels. This finding aligns with the study by Inocian et al., which reported that the pandemic led to an increase in sedentary behavior among nurses, primarily due to high stress levels, longer shifts, and a lack of time for physical self-care [20]. However, pediatric nurses appeared to maintain higher levels of physical activity, with a notable increase in daily exercise compared to general hospital nurses. It is possible that pediatric nurses recognized the central role of physical activity as a coping strategy for enhancing their physical and mental well-being. This finding is consistent with the study by Pit and Hansen, which highlighted the positive impact of physical activity in reducing occupational stress and psychological distress among healthcare professionals, mitigating the phenomenon of presenteeism [21]. The present study found that both groups of nurses appeared to have reduced their frequency of social outings since the beginning of the pandemic. General nurses reported fewer social interactions and an increase in the "1-2 days per week" category, which rose to 40.6% (n = 59). Additionally, a higher percentage of general hospital nurses reported not going out at all in the past two weeks compared to pediatric nurses, suggesting a greater level of social restriction among general hospital nurses. The study by Anger et al. documented that nurses experiencing high levels of stress and depression were more likely to limit their social activities, supporting our finding that nurses working in general hospitals reported a higher level of social isolation compared to pediatric nurses [22]. Although there is no specific literature comparing social contact restrictions between pediatric and general hospital nurses, research indicates that nurses across all hospital departments experienced significant stress due to increased workload, extended working hours, and the emotional toll of patient care during the pandemic [23]. This context likely led to increased social isolation as a precautionary measure against infection and as a response to physical and mental exhaustion. In contrast, pediatric nurses, while still experiencing anxiety and stress, faced different challenges related to caring for children and supporting families, which may have influenced their risk perceptions and social behaviors differently [24]. These variations in the type and severity of stressors between nursing groups may have indirectly affected their social interactions during the pandemic.

Strengths and limitations

This study revealed significant differences between pediatric and general hospital nurses. It highlights the substantial psychological and physical impact of the COVID-19 pandemic on both pediatric and general hospital nurses. However, certain limitations must be acknowledged. First, the study did not differentiate between pediatric nurses who continued working in pediatric wards and those reassigned to general hospitals. This lack of distinction may have affected the analysis of the specific stressors faced by each group. Additionally, the use of self-reported data may introduce potential biases or inaccuracies, as participants’ perceptions of their physical and mental health may not perfectly align with objective measures. One more important limitation is the use of a convenience sampling method, which may affect the generalizability of the findings. Future studies should consider employing randomized or stratified sampling techniques to enhance representativeness.

## Conclusions

Overall, the COVID-19 pandemic had a profound impact on the physical and mental health of both pediatric and general hospital nurses. Our study reveals that pediatric nurses experienced greater physical health deterioration than their counterparts in general hospitals, likely due to redeployment to more physically demanding environments and understaffing in pediatric units. These findings highlight the urgent need for targeted interventions to support the well-being of nurses in both specialties. Addressing issues such as adequate staffing, reducing physical workloads, and implementing tailored support systems can help mitigate the long-term effects of the pandemic.

To enhance resilience and reduce stress, healthcare institutions and policymakers should prioritize evidence-based workplace interventions, such as structured stress management programs, peer support networks, and resilience training workshops. Implementing flexible scheduling, mental health counseling services, and targeted wellness programs can significantly improve nurses’ well-being. Additionally, ergonomic modifications in pediatric settings, such as proper lifting equipment and workstation adjustments, could help reduce physical strain. Institutional policies should also focus on burnout prevention strategies, such as workload redistribution and proactive mental health monitoring. The lessons learned from this healthcare crisis should drive policies aimed at enhancing resilience, improving working conditions, and ensuring that nurses are better equipped to handle future challenges. Strengthening occupational health policies at a national level and integrating long-term mental health resources into hospital structures will be crucial in sustaining a healthier workforce.
